# Area-Based Socioeconomic Inequalities in Colorectal Cancer Survival in Germany: Investigation Based on Population-Based Clinical Cancer Registration

**DOI:** 10.3389/fonc.2020.00857

**Published:** 2020-05-29

**Authors:** Lina Jansen, Gundula Behrens, Isabelle Finke, Werner Maier, Michael Gerken, Ron Pritzkuleit, Bernd Holleczek, Hermann Brenner, for the German Cancer Survival Working Group

**Affiliations:** ^1^Division of Clinical Epidemiology and Aging Research, German Cancer Research Center (DKFZ), Heidelberg, Germany; ^2^Medical Faculty Heidelberg, University of Heidelberg, Heidelberg, Germany; ^3^Helmholtz Zentrum München—German Research Center for Environmental Health (GmbH), Institute of Health Economics and Health Care Management, Neuherberg, Germany; ^4^Tumor Center—Institute for Quality Management and Health Services Research, University of Regensburg, Regensburg, Germany; ^5^Cancer Registry of Schleswig-Holstein, Lübeck, Germany; ^6^Saarland Cancer Registry, Saarbrücken, Germany; ^7^Division of Preventive Oncology, German Cancer Research Center (DKFZ), National Center for Tumor Diseases (NCT), Heidelberg, Germany; ^8^German Cancer Consortium (DKTK), German Cancer Research Center, Heidelberg, Germany

**Keywords:** colorectal cancer, area-based socioeconomic deprivation, survival, treatment, Germany

## Abstract

**Background:** Socioeconomic inequalities in colorectal cancer survival have been observed in many countries. To overcome these inequalities, the underlying reasons must be disclosed.

**Methods:** Using data from three population-based clinical cancer registries in Germany, we investigated whether associations between area-based socioeconomic deprivation and survival after colorectal cancer depended on patient-, tumor- or treatment-related factors. Patients with a diagnosis of colorectal cancer in 2000–2015 were assigned to one of five deprivation groups according to the municipality of the place of residence using the German Index of Multiple Deprivation. Cox proportional hazards regression models with various levels of adjustment and stratifications were applied.

**Results:** Among 38,130 patients, overall 5-year survival was 4.8% units lower in the most compared to the least deprived areas. Survival disparities were strongest in younger patients, in rectal cancer patients, in stage I cancer, in the latest period, and with longer follow-up. Disparities persisted after adjustment for stage, utilization of surgery and screening colonoscopy uptake rates. They were mostly still present when restricting to patients receiving treatment according to guidelines.

**Conclusion:** We observed socioeconomic inequalities in colorectal cancer survival in Germany. Further studies accounting for potential differences in non-cancer mortality and exploring treatment patterns in detail are needed.

## Introduction

Colorectal cancer (CRC) is the third most common cancer worldwide with more than 1.8 million cases in 2018 (1). In Germany, about 61,000 persons were diagnosed with CRC in 2014, (2) and 5-year relative survival for CRC was estimated to be 64% for patients diagnosed in 2007–2010 in Germany (3).

Previous studies have elucidated socioeconomic inequalities in survival after CRC (4, 5). These inequalities were observed for socioeconomic status measures on individual as well as area level and in countries with and without a universal health insurance system. In Germany, individual and area-based socioeconomic inequalities in cancer survival have been rarely investigated (6–8). In a multi-center cohort study including 1,633 cancer patients, individual-level higher income and better vocational training was associated with better cancer prognosis (6). Three studies on the association of area-based socioeconomic deprivation and cancer survival in Germany have been conducted (9–11). The first study was conducted more than 25 years ago and reported shorter survival after CRC for patents living in more deprived communities in the state Saarland (9). In the first multi-state study, area-based socioeconomic deprivation was measured on the district level (median population: ≈126,000 residents) and was significantly associated with relative survival for many cancer sites (10). For CRC, 5-year relative survival in 2002–2006 decreased from 64.5% for patients living in the least deprived regions to 59.6% for patients living in the most deprived regions. Differences persisted after adjustment for tumor stage. In a recent study on lung cancer, area-based socioeconomic inequalities in survival were reported, which were stronger in earlier stages (11).

While identification and quantification of socioeconomic inequalities in cancer survival is a starting point, knowledge on the underlying reasons is indispensable to overcome these inequalities. Proposed explanations for deprivation-associated inequalities include differences in patient or tumor characteristics and variations in quality and utilization of as well as compliance with medical care (12, 13). Previous studies from several countries have reported that CRC patients living in more deprived areas or having a lower socioeconomic status received less often surgery and adjuvant or neoadjuvant treatment (5). Furthermore, they participate less often in CRC screening programs (14). However, for Germany, it has not been investigated yet whether inequalities in CRC survival can be explained by differences in screening or cancer care.

Here, we investigated the association between area-based socioeconomic deprivation and CRC survival in Germany by using data from population-based clinical cancer registries. As individual data on socioeconomic status measures, such as income or level of education, cannot be linked to cancer registry data in Germany, we use a multi-dimensional area-based deprivation measure, which is based on aggregated data from official statistics. For the first time, it was possible to link this measure to cancer registry data on municipality level (median population: ≈2,200 residents, range: 128–523,058). Using this linked dataset, we investigated whether and to what extent deprivation-associated survival inequalities are present in Germany and whether these associations can be explained by demographic or clinical factors.

## Materials and Methods

### Study Population

Data was provided from three regional population-based clinical cancer registries located in the South and East of Germany (Regensburg, Erfurt and Dresden) and covering parts of the German states Bavaria, Thuringia and Saxony, respectively (≈3.91 million residents in 2013) (15). The registries covered a population with 2.06 million, 707.635 and 1.15 million inhabitants, of whom 135,520 (6.6%), 204,994 (29.0%), and 523,058 (73.9%) lived in the central cities Regensburg, Erfurt and Dresden and the remaining population lived in 447, 232, and 97 municipalities with median population of 2,629, 743, and 3,381 inhabitants, respectively (15).

Patients aged 15 years or older who were resident in the catchment areas of one of the above-mentioned registries and were diagnosed with a primary tumor of the colon or rectum (ICD 10 C18-C20) in 2000–2015 were eligible for the analysis. If a patient had multiple CRC diagnoses within 3 months, information from these diagnoses were combined (first date of diagnosis, highest stage and grade). If the time interval between diagnoses was larger than 3 months, only information from the first diagnosis was included. Death certificate or autopsy only cases, cases where no deprivation score could be linked and cases with no or 0 months of follow-up were excluded (Supplementary Figure 1). Patients were censored at date of last contact if they were lost to follow-up or at the end of 2015 if they were still alive.

### Area-Based Socioeconomic Deprivation

Area-based socioeconomic deprivation of the patients was assessed using the German Index of Multiple Deprivation (GIMD) on municipality level (16). The GIMD is based on data of official statistics and consists of seven single domains (income, employment, education, municipality revenue, social capital, environment, and security deprivation), and a composite index. Two editions of this deprivation index are available based on data from 2006 and from 2010 (or the next year available), respectively. For the composite index, deprivation quintiles were computed specifically for this analysis using the underlying population of the included municipalities to have comparable sample sizes across groups (2006: 792 municipalities, median population: 2205; 2010: 779 municipalities, median population: 2189; Table 2) (15). In addition, region-specific deprivation quintiles were computed for the catchment area of each registry. In these analyses, the large cities Dresden and Erfurt were assigned a separate group. The range of GIMD values in each quintile are shown in Table 1 and a map illustrating the distribution of the overall quintiles based on the GIMD 2010 is provided in Supplementary Figure 2. The quintiles were assigned to the patient according to the municipality of residence at the time of diagnosis using the version that is closer to the date of diagnosis.

**Table 1:** Range of German index of multiple deprivation values in each quintile.

	**Range of GIMD values**					
	**Deprivation quintiles**					**Cities**
**GIMD version and registry**	**Q1 (least deprived)**	**Q2**	**Q3**	**Q4**	**Q5 (most deprived)**	**Dresden/Erfurt^**a**^**
GIMD 2006						
All registries	4.73–17.00	17.01–17.75	17.80–21.53	21.55–26.06	26.08–57.20	–
Dresden	8.90–17.28	18.53–22.65	22.67–25.09	26.03–32.57	32.99–46.82	17.59
Erfurt	9.83–20.13	20.34–24.28	24.33–25.96	26.14–27.79	27.96–57.20	20.07
Regensburg	4.73–15.22	15.23–17.55	17.55–21.31	21.33–25.56	25.70–39.57	–
GIMD 2010						
All registries	4.94–17.86	17.87–24.10	24.15–26.06	26.17–30.64	30.65–60.44	–
Dresden	10.37–20.08	20.33–26.98	27.12–31.86	32.02–38.87	39.05–47.24	26.06
Erfurt	12.09–24.38	24.39–29.44	29.51–32.67	32.38–36.93	37.22–60.44	26.17
Regensburg	4.94–14.20	14.34–18.24	18.25–22.46	22.59–26.30	26.32–43.89	–

*Q, quintile; GIMD, German index of multiple deprivation*.

a *for the cancer registries Dresden and Erfurt, the cities Dresden and Erfurt were classified separately, as they would otherwise dominate the classification of the quintiles*.

**Table 2:** Characteristics of colorectal cancer patients by socioeconomic deprivation quintile.

		**Deprivation quintile**					
**Factor**	**Total**	**Least deprived (Q1)**	**Q2**	**Q3**	**Q4**	**Most deprived (Q5)**	***P*-value^**f**^**
	***N* = 38,130**	***N* = 7,453 (19.5%)**	***N* = 8,123 (21.3%)**	***N* = 7,710 (20.2%)**	***N* = 7,223 (18.9%)**	***N* = 7,622 (20.0%)**	
Registry^a^							
Dresden	11,284 (29.6)	925 (12.4)	3,346 (41.2)	2,854 (37.0)	1,289 (17.8)	2,870 (37.7)	<0.0001
Erfurt	6,783 (17.8)	215 (2.9)	438 (5.4)	1,717 (22.3)	2,178 (30.2)	2,235 (29.3)	
Regensburg	20,063 (52.6)	6,312 (84.7)	4,339 (53.4)	3,139 (40.7)	3,756 (52.0)	2,517 (33.0)	
Sex							
Male	22,214 (58.3)	4,522 (60.7)	4,646 (57.2)	4,465 (57.9)	4,213 (58.3)	4,368 (57.3)	<0.0001
Female	15,916 (41.7)	2,931 (39.3)	3,477 (42.8)	3,245 (42.1)	3,010 (41.7)	3,254 (42.7)	
Age at diagnosis							
15–54	4,262 (11.2)	1,006 (13.5)	864 (10.6)	821 (10.6)	799 (11.1)	772 (10.1)	<0.0001
55–64	7,854 (20.6)	1,574 (21.1)	1,683 (20.7)	1,500 (19.5)	1,513 (20.9)	1,584 (20.8)	
65–69	6,051 (15.9)	1,119 (15.0)	1,292 (15.9)	1,244 (16.1)	1,145 (15.9)	1,251 (16.4)	
70–74	6,816 (17.9)	1,273 (17.1)	1,446 (17.8)	1,398 (18.1)	1,251 (17.3)	1,448 (19.0)	
75–79	6,198 (16.3)	1,234 (16.6)	1,286 (15.8)	1,246 (16.2)	1,195 (16.5)	1,237 (16.2)	
80+	6,949 (18.2)	1,246 (16.7)	1,552 (19.1)	1,501 (19.5)	1,320 (18.3)	1,330 (17.4)	
Mean (std)	69.0 (11.5)	68.2 (11.8)	69.2 (11.6)	69.5 (11.5)	69.1 (11.4)	69.1 (11.1)	
Year of diagnosis							
2000–2007	18,394 (48.3)	3,330 (44.7)	3,904 (48.0)	3,602 (46.8)	3,650 (50.6)	3,908 (51.3)	<0.0001
2008–2015	19,736 (51.8)	4,122 (55.3)	4,219 (51.9)	4,108 (53.2)	3,573 (49.5)	3,714 (48.7)	
Tumor location							
Colon	22,946 (60.2)	4,418 (59.3)	5,000 (61.6)	4,753 (61.6)	4,278 (59.2)	4,497 (59.0)	<0.0001
Rectum^b^	14,392 (37.6)	2,868 (38.5)	2,969 (36.6)	2,765 (35.9)	2,799 (38.8)	2,962 (38.9)	
Both	821 (2.2)	166 (2.2)	154 (1.9)	192 (2.5)	146 (2.0)	163 (2.1)	
Stage^c^							
I	7,073 (21.6)	1,244 (19.9)	1,607 (22.4)	1,501 (22.2)	1,257 (21.0)	1,464 (22.1)	<0.0001
II	8,898 (27.1)	1,610 (25.7)	1,933 (26.9)	1,878 (27.7)	1,679 (28.1)	1,798 (27.1)	
III	9,182 (28.0)	1,898 (30.3)	2,013 (28.1)	1,787 (26.4)	1,641 (27.4)	1,843 (27.8)	
IV	7,663 (23.4)	1,511 (24.1)	1,621 (22.6)	1,609 (23.7)	1,404 (23.5)	1,518 (22.9)	
Grading^d^							
Low	26,689 (73.8)	5,636 (80.0)	5,637 (73.4)	5,195 (71.4)	5,034 (72.5)	5,187 (71.8)	<0.0001
High	9,482 (26.2)	1,408 (20.0)	2,047 (26.6)	2,083 (28.6)	1,911 (27.5)	2,033 (28.2)	
Surgery							
No	3,548 (9.3)	692 (9.3)	720 (8.9)	711 (9.2)	690 (9.6)	735 (9.6)	<0.0001
Yes	34,582 (90.7)	6,760 (90.7)	7,403 (91.1)	6,999 (90.8)	6,533 (90.4)	6,887 (90.4)	
R classification^e^							
R0	29,418 (91.9)	5,849 (94.7)	6,440 (92.4)	5,924 (90.8)	5,481 (91.3)	5,724 (90.3)	<0.0001
R1/2	2,592 (8.1)	330 (5.3)	533 (7.6)	597 (9.1)	520 (8.6)	612 (9.7)	

*Q, Quintile; std, standard deviation*.

a *included regions: Upper Palatinate, Lower Bavaria, and the districts of Erfurt, Eisenach, Wartburg, Unstrut-Hainich, Gotha, Sömmerda, Ilm, Weimarer Land, Meisen, Saxon Switzerland-East Ore Mountains, and Bautzen*.

b *including rectal (ICD-10 C20) and rectosigmoid (C19) tumors*.

c *missing stage at diagnosis: n = 5,314 (13.9%)*.

d *missing grading: n = 1,959 (5.1%)*.

e *missing R classification among patients with surgery: n = 2,572 (7.4%)*.

f *p-value from Chi-square test comparing the distribution of the factor across deprivation quintiles*.

### Covariates

Information on age at diagnosis, sex, place of residence, month and year of diagnosis, cancer site and stage, tumor grade, primary treatment (surgery/chemotherapy/radiotherapy), resection margin (R0: no cancer cells seen microscopically at the primary tumor site after surgery, R1/2: cancer cells present microscopically or macroscopically at the primary tumor site or regional lymph nodes), follow-up time in months and vital status at end of follow-up (alive, dead) were extracted from the cancer registry datasets. During data quality checks, strong differences in chemotherapy and radiotherapy utilization proportions across registries and calendar periods were detected. It could not be ruled out that those differences were due to differences in the completeness of treatment registration, which might result in biases in regional analyses. However, if the treatment variable explicitly indicated that a specific therapy was actually given, this information was expected to be reliable. We therefore used treatment for subgroup analyses by restricting the sample to those patients with information on provided specific treatments.

As information on participation in CRC screening was not available on individual level, the estimated screening colonoscopy participation rate on district level was retrieved from health care claims data (17). Data was available for 2008–2011 and assigned to the patient according to the district of residence at the time of diagnosis using the version that is closest to the date of diagnosis.

### Statistical Analysis

Demographic and clinical characteristics by area-based socioeconomic deprivation quintile were described and distributions were compared using Chi-square tests. Before multivariable analyses, missing values were imputed by applying Multivariate Imputation by Chained Equation (Supplementary Material) (18). The distribution of imputed variables was comparable before and after imputation (Supplementary Table 1).

Median follow-up time was estimated with the reverse Kaplan-Meier method (19). Overall survival (death from any cause) after CRC diagnosis was estimated for each deprivation quintile using Kaplan-Meier curves.

To test whether inequalities in cancer survival can be explained by demographic, tumor-related or treatment factors, Cox proportional hazard regression models were computed with various pre-specified levels of adjustment. When associations weaken or disappear when adjusting for specific factors, for example for stage, it can be hypothesized that these factors at least partly explain the socioeconomic survival difference and can be targeted in interventions to overcome these differences. The base model included adjustment for age at diagnosis, sex, year of diagnosis, cancer site and grade. The second model additionally included cancer stage. The third model, which was pre-defined as main model and used in all stratified analyses, additionally adjusted for surgery. In the fourth model, further adjustment for the utilization proportions of screening colonoscopy was added. Main results were visualized in adjusted survival curves.

To test whether socioeconomic inequalities in cancer survival vary across subgroups, pre-specified stratified analyses by age at diagnosis, sex, year of diagnosis, cancer site, and cancer stage and by restricting the length of follow-up were conducted.

Adjustment for treatment factors was not possible due to the data quality issues described above. To nonetheless test whether socioeconomic inequalities in cancer survival are affected by treatment administration, we repeated the analyses in subgroups of patients who received cancer treatment according to recommendations in treatment guidelines.

To test whether socioeconomic inequalities in survival were present and comparable in each of the three included regions, analyses were repeated within each region using region-specific GIMD quintiles.

In sensitivity analyses, the city of Dresden, which was classified in the GIMD quintile 2 in 2000–2008 and quintile 3 in 2009–2015 in the main analyses, was classified as separate category. This analysis was the only one added *post-hoc*, but was necessary to investigate the impact of this large city on the associations over all regions.

The proportional hazard assumption was verified for each model by exploring Schoenfeld residuals. All tests were two-sided with α = 0.05 and no multiple comparison correction. Multiple imputation was conducted in R (Version 3.5.2). All other analyses were conducted in SAS 9.4 (Cary, NC: SAS Institute Inc.).

## Results

### Patient Characteristics

In total, 38,130 patients with a diagnosis of CRC in 2000–2015 were included. Mean age at diagnosis was 69 years (Table 2). Most of the patients were living in the catchment area of the cancer registry Regensburg (53%), were male (58%), had colon cancer (60%), stage II (27%), or III disease (28%) and a tumor of low grade (74%). Surgery was conducted in 91% of the patients. Of these patients, 92% had no residual tumor after surgery. A provided chemotherapy and radiotherapy were administered registered in 44 and 17% of the patients, respectively. Despite statistically significant differences across deprivation groups for all patient and tumor characteristics, no consistent pattern across the groups were observed except for more low grade cancers (80 vs. 72% in the least and most deprived group) and more R0 resections (95 vs. 90%) in the least deprived group.

### Survival in the Total Group

**Figure 1:**
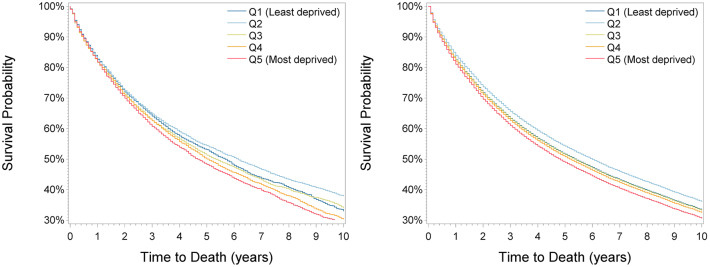
Kaplan-Meier **(Left)** and adjusted survival curves **(Right)** by deprivation quintiles for all patients.

During a median follow-up time of 6.25 years (25th percentile: 3.25 years, 75th percentile: 10 years), 19,277 patients (50%) died. Survival decreased with increasing deprivation (Figure 1) except for a higher survival in the second least deprived group (Q2), with 5-year survival of 53.1% (95% confidence interval: 51.8–54.4), 54.4% (53.2–55.6), 51.3% (50.4–52.6), 50.1% (48.8–51.4), and 48.3% (47.0–49.5) from the least to the most deprived group. After adjustment for sex, age at diagnosis, period, cancer site, and cancer grade (basis model), survival was highest in the second least deprived group (hazard ratio: 0.86 (95% confidence interval: 0.82–0.90) compared to the least deprived group) and lowest in the most deprived group (1.08 (1.03–1.13), Table 3). When additionally adjusting for stage, associations were almost comparable [second least deprived group: 0.90 (0.86–0.94); most deprived group: 1.11 (1.06–1.16)]. Additional adjustment for surgery had no effect on the estimates. Direct adjusted survival curves derived from the fully adjusted model are shown in Figure 1. Adjusted 5-year survival estimates are shown in Supplementary Tables 2, 3, respectively, and illustrate that the difference in 5-year survival between the least and most deprived group changed only marginally by stage adjustment from −2.5% units to −2.7% units and kept constant after adjustment for surgery.

**Table 3:** Association of socioeconomic deprivation and cancer survival and adjusted 5-year observed survival estimates, overall with different levels of adjustment and stratified by patient and tumor characteristics with full adjustment.

**Subgroup (Model^**a**^)**	**Events [N (%)]**	**Deprivation quintile [Hazard ratio (95% confidence interval)]**					**5-year survival (standard error)**^****e****^		
		**Q1(least deprived)**	**Q2**	**Q3**	**Q4**	**Q5 (most deprived)**	**Q1 (%)**	**Q5 (%)**	**Q5–Q1 (% units)**
All (Basic model)	19,227 (50.4)	1.00 (ref)	**0.86** ** (0.82–0.90)**	0.97 (0.93–1.01)	1.04 (0.99–1.09)	**1.08** ** (1.03–1.13)**	51.0	48.5	−2.5
All (+Stage)	19,227 (50.4)	1.00 (ref)	**0.90** ** (0.86–0.94)**	1.01 (0.96–1.05)	1.04 (0.99–1.09)	**1.11** ** (1.06–1.16)**	51.7	49.0	−2.7
All (+ Surgery)	19,227 (50.4)	1.00 (ref)	**0.90** ** (0.86–0.95)**	1.01 (0.96–1.05)	1.03 (0.99–1.08)	**1.11** ** (1.06–1.16)**	51.7	49.0	−2.7
Exclusion of Dresden^b^	15,023 (50.6)	1.00 (ref)	0.97 (0.92–1.03)	1.03 (0.98–1.09)	1.03 (0.98–1.08)	**1.11** ** (1.06–1.16)**	51.5	48.8	−2.7
Male	11,101 (50.0)	1.00 (ref)	**0.88** ** (0.83–0.94)**	1.00 (0.94–1.06)	1.02 (0.96–1.08)	**1.10** ** (1.03–1.17)**	49.7	47.3	−2.4
Female	8,126 (51.1)	1.00 (ref)	0.94 (0.87–1.01)	1.03 (0.95–1.10)	1.06 (0.98–1.15)	**1.13** ** (1.05–1.22)**	54.4	51.3	−3.1
Age 15–64 years	4,456 (36.8)	1.00 (ref)	0.91 (0.83–1.00)	0.96 (0.87–1.06)	1.09 (0.99–1.20)	**1.16** ** (1.05–1.27)**	64.6	61.6	−3.0
Age 65+ years	14,771 (56.8)	1.00 (ref)	**0.90** ** (0.86–0.95)**	1.01 (0.96–1.07)	1.02 (0.96–1.08)	**1.07** ** (1.01–1.13)**	45.2	43.2	−2.0
Period 2000–2007	11,615 (63.2)	1.00 (ref)	**0.83** ** (0.78–0.88)**	1.01 (0.95–1.08)	0.97 (0.91–1.03)	1.05 (0.99–1.11)	50.7	49.4	−1.3
Period 2008–2015	7,612 (38.6)	1.00 (ref)	1.03 (0.96–1.11)	0.99 (0.92–1.06)	**1.13** ** (1.05–1.22)**	**1.19** ** (1.11–1.28)**	53.1	48.5	−4.6
Colon^c^	11,559 (50.4)	1.00 (ref)	**0.88** ** (0.83–0.93)**	1.01 (0.95–1.07)	0.99 (0.93–1.06)	**1.06** ** (1.00–1.13)**	52.8	51.2	−1.6
Rectum^c^	7,201 (50.1)	1.00 (ref)	0.93 (0.86–1.01)	1.02 (0.94–1.11)	**1.09** ** (1.01–1.18)**	**1.18** ** (1.10–1.28)**	50.1	45.6	−4.5
Stage I	2,706 (31.1)	1.00 (ref)	**0.79** ** (0.69–0.89)**	0.98 (0.86–1.11)	1.13 (0.99–1.28)	**1.18** ** (1.04–1.34)**	72.3	68.8	−3.5
Stage II	4,166 (41.3)	1.00 (ref)	**0.84** ** (0.76–0.92)**	0.89 (0.81–0.99)	0.95 (0.86–1.05)	**1.12** ** (1.02–1.24)**	62.7	59.6	−3.1
Stage III	4,791 (47.1)	1.00 (ref)	0.95 (0.86–1.04)	**1.11** ** (1.01–1.22)**	**1.16** ** (1.05–1.27)**	**1.12** ** (1.02–1.23)**	55.7	52.4	−3.3
Stage IV	7,564 (82.3)	1.00 (ref)	0.96 (0.89–1.03)	0.99 (0.92–1.07)	0.96 (0.88–1.04)	1.02 (0.95–1.10)	13.8	13.2	−0.6
FU length: 3 months^d^	2,426 (6.4)	1.00 (ref)	1.01 (0.89–1.15)	1.11 (0.98–1.27)	1.07 (0.94–1.22)	1.07 (0.94–1.22)	Na	Na	Na
FU length: 1 year^d^	6,503 (17.1)	1.00 (ref)	1.01 (0.93–1.09)	1.03 (0.95–1.12)	1.00 (0.92–1.08)	1.03 (0.95–1.12)	Na	Na	Na
FU length: 5 years^d^	15,649 (41.0)	1.00 (ref)	0.97 (0.92–1.02)	1.03 (0.98–1.09)	1.03 (0.98–1.09)	**1.12** ** (1.06–1.17)**	52.4	49.6	−2.8
FU length: 10 years^d^	18,630 (48.9)	1.00 (ref)	**0.93** ** (0.89–0.98)**	1.01 (0.96–1.05)	1.03 (0.98–1.08)	**1.11** ** (1.06–1.17)**	51.8	49.0	−2.8

ref, Reference; Q, quintile; Na, not applicable as follow-up was restricted to <5 years; Significant hazard ratios (p < 0.05) are printed in bold;

a *for the overall analyses, three models were used. The basic model includes an adjustment for sex, age, period, cancer site and cancer grade. In the second model, stage and in the third model, surgery was added. In all stratified analyses, model three was used (after omitting the stratification factor)*.

b *the city Dresden was the largest city in the covered area, comprising 13.4% of the total underlying study population*.

c patients with a diagnosis of both colon and rectum cancer within 3 months were excluded. Rectum includes rectal (ICD-10 C20) and rectosigmoid (C19) tumors.

d follow-up length was restricted to a certain time window. Patients dying after this time were censored at the end of the time window.

e *five-year overall survival derived from the direct adjusted survival curves from the adjusted Cox proportional hazard regression model*.

### Survival in Patient Subgroups

**Figure 2:**
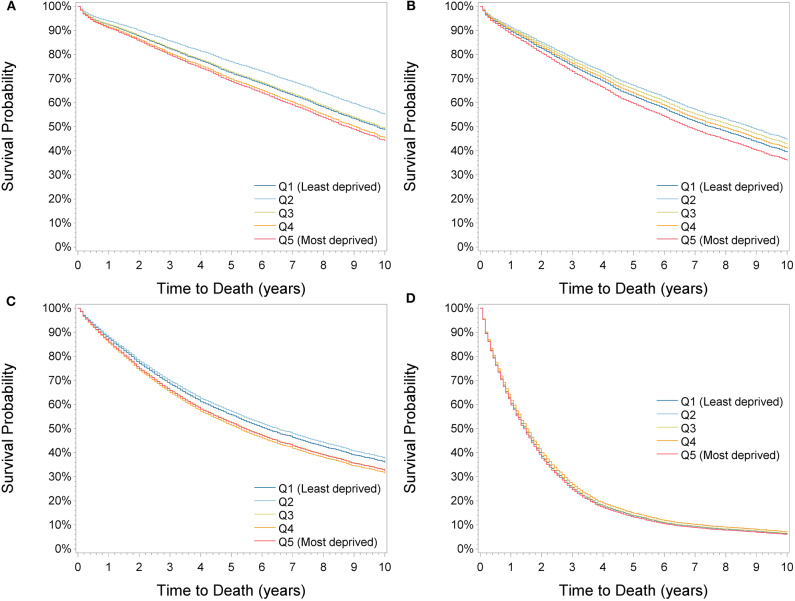
Adjusted survival curves by deprivation quintiles for stage I **(A)**, stage II **(B)**, stage III **(C)**, and stage IV **(D)** patients.

Using the fully adjusted model, subgroup analyses by sex, age at diagnosis, calendar period, cancer site, stage and length of follow-up were conducted (Table 3, Supplementary Table 2) and adjusted survival curves by cancer stage were derived (Figure 2). A general pattern observed in many subgroups was that survival was highest in the second least deprived group. This association was particularly pronounced in stage I patients [0.79 (0.78–0.88)] and in the period 2000–2007 [0.83 (0.78–0.88)]. Classifying the city of Dresden as a separate deprivation category completely resolved the survival advantage in the second least deprived group and showed highest survival in Dresden in all subgroups of patients except in those with stage IV cancer and when restricting the follow-up length to 3 months or 1 year (Supplementary Table 3). Consequently, when excluding Dresden in the overall analysis, the estimated HRs of patients with stage II cancer dropped from 0.97 (0.92–1.03) to 0.90 (0.86–0.95) in the second most affluent group, while the other estimates changed only slightly.

Survival was significantly lower in the most deprived compared to the least deprived group in all subgroups except for the period 2000–2007, in stage IV cancer patients and when restricting the follow-up length to 3 months or 1 year (Table 3, Supplementary Table 2). Associations were stronger in patients aged 15–64 years [1.16 (1.05–1.27)] compared to patients aged 65+ years [1.07 (1.01–1.13)] and in patients with rectal [1.18 (1.10–1.28)] compared to colon tumors [1.06 (1.00–1.13)]. Differences in 5-year survival between the least and most deprived group were largest in 2008–2015 (−4.6% units), in rectal cancer patients (−4.5% units) and in patients with stage I tumors (−3.5% units).

Overall, no gradual trend in survival from the least to the most deprived group was observed (Table 3, Supplementary Table 2). Furthermore, a comparison of the third (Q3) and second (Q2) most deprived group to the least deprived group showed significant associations for few subgroups only: Survival was significantly lower in the second most deprived group compared to the least deprived group in 2008–2015 [1.13 (1.05–1.22)], in rectal cancer patients [1.09 (1.01–1.18)], and in patients with stage III cancer [1.16 (1.05–1.27)]. Patients in the third group had significantly lower survival in patients with stage III cancer only [1.11 (1.01–1.22)].

### Survival After Adjustment for Screening Utilization

As differences in the utilization of colonoscopy screening between deprivation groups might have had an impact on the association between survival and deprivation, analyses were repeated with additional adjustment for utilization proportions of colonoscopy screening in the districts of the place of residence of the patient (Supplementary Table 4). This adjustment weakened the previously observed lower survival in the most deprived group only slightly [with adjustment: 1.09 (1.04–1.15); without adjustment: 1.11 (1.06–1.16)] and no consistent pattern of change in the subgroup analyses was observed.

### Survival in Subgroups by Cancer Treatment

To assess the impact of treatment on the association of deprivation with survival, subgroup analyses by treatment were conducted (Table 4, Supplementary Table 5). Again, a survival advantage in the second least deprived group was observed in some subgroup analyses, which could be attributed to a better survival in the city of Dresden (Supplementary Table 6) and could not be explained by restriction to patients who received specific treatments. For patients with stage I and stage II-III CRC, the significant survival disadvantage in the second most deprived and most deprived group remained after restriction to patients who had surgery (stage I) and patients who had surgery and received chemotherapy (stage II–III). In stage III colon cancer, associations did not change consistently when restricting subsequently to patients with R0 surgery and with chemotherapy. In stage II–III rectal cancer, the significant survival disadvantage in the second most deprived group resolved [from 1.12 (1.00–1.26) to 0.95 (0.76–1.19)] and in the most deprived group weakened [from 1.18 (1.05–1.32) to 1.13 (0.91–1.41)] when restricting to patients who received neoadjuvant radiotherapy. However, the difference in 5-year relative survival between the most and least deprived group did not decrease (−4.9% units compared to −4.8% units). In general, sample sizes were small in subgroup analyses resulting in large confidence intervals, which hampered the interpretation of the results.

**Table 4:** Association of socioeconomic deprivation and observed survival for subgroups according to received treatment.

**Subgroup**	**Events [N (%)]**	**Deprivation quintile [Hazard ration (95% confidence interval)]**^****a****^					**5-year survival (standard error)**^****b****^		
		**Q1(least deprived)**	**Q2**	**Q3**	**Q4**	**Q5(most deprived)**	**Q1 (%)**	**Q5 (%)**	**Q5–Q1 (% units)**
Stage I	2,706 (31.1)	1.00 (ref)	**0.79 (0.70–0.90)**	0.99 (0.87–1.13)	**1.14 (1.01–1.30)**	**1.19 (1.05–1.35)**	74.3	70.7	−3.6
+ with surgery	2,524 (30.0)	1.00 (ref)	**0.78 (0.68–0.88)**	0.99 (0.87–1.13)	**1.15 (1.01–1.31)**	**1.20 (1.06–1.37)**	75.3	71.5	−3.8
Stage II&III	8,957 (47.1)	1.00 (ref)	**0.90 (0.84–0.96)**	1.02 (0.96–1.10)	**1.09 (1.02–1.17)**	**1.15 (1.07–1.23)**	60.6	56.7	−3.9
+ surgery	8,321 (42.9)	1.00 (ref)	**0.90 (0.84–0.97)**	1.02 (0.95–1.10)	**1.09 (1.01–1.17)**	**1.15 (1.07–1.23)**	61.9	58.1	−3.8
+ chemotherapy	3,640 (38.4)	1.00 (ref)	0.91 (0.82–1.02)	1.07 (0.96–1.19)	**1.13 (1.02–1.26)**	**1.17 (1.05–1.30)**	65.8	61.5	−4.3
Stage III colon cancer	2,726 (47.4)	1.00 (ref)	0.94 (0.83–1.07)	**1.14 (1.01–1.29)**	**1.17 (1.03–1.34)**	1.13 (0.99–1.28)	59.2	55.7	−3.5
+ with R0 surgery	2,280 (47.7)	1.00 (ref)	0.95 (0.83–1.08)	1.13 (0.99–1.30)	1.14 (0.99–1.31)	1.10 (0.96–1.26)	61.6	59.0	−2.6
+ with chemotherapy	1,142 (36.3)	1.00 (ref)	0.88 (0.73–1.06)	1.19 (0.99–1.43)	1.11 (0.92–1.35)	1.18 (0.98–1.43)	68.6	64.2	−4.4
Stage II & III rectal cancer	3,102 (45.3)	1.00 (ref)	0.93 (0.83–1.04)	1.04 (0.93–1.17)	**1.12 (1.00–1.26)**	**1.18 (1.05–1.32)**	55.7	50.8	−4.9
+ radiotherapy	1,738 (40.1)	1.00 (ref)	0.87 (0.75–1.02)	1.06 (0.91–1.24)	**1.16 (1.00–1.34)**	**1.15 (1.00–1.34)**	60.1	56.4	−3.7
+ neoadjuvant radiotherapy	751 (30.2)	1.00 (ref)	0.82 (0.66–1.02)	0.98 (0.79–1.22)	0.95 (0.76–1.19)	1.13 (0.91–1.41)	60.9	56.1	−4.8

*Ref, reference; Q, quintile; Significant hazard ratios (p < 0.05) are printed in bold*.

a *adjusted for age, sex, period, cancer site (for overall analyses), cancer grade and cancer stage (for stage II&III analyses)*.

b *five-year overall survival derived from the direct adjusted survival curves from the adjusted Cox proportional hazard regression model*.

### Survival Within the Included Regions

Using region-specific deprivation quintiles, the association between deprivation and survival within the catchment areas of the cancer registries was investigated (Table 5, Supplementary Table 7). In these analyses, the two large cities, Dresden and Erfurt, were classified separately. Compared to the least deprived group in the region of Dresden, survival was significantly lower in the most deprived group [1.18 (1.06–1.32)] with 4.3% units lower 5-year survival and significantly higher in the city Dresden [0.82 (0.75–0.90)] with 4.8% units higher 5-year survival. In the regions Regensburg and Erfurt, deprivation was not significantly associated with survival.

**Table 5:** Association of socioeconomic deprivation and observed survival and adjusted 5-year survival estimates for each single registry using deprivation quintiles derived within the catchment area of the registry.

**Cancer registry**	**Deprivation quintile [Hazard ration (95% confidence interval)]**^****a****^						**5-year survival (standard error)**^****c****^		
	**Q1(least deprived)**	**Q2**	**Q3**	**Q4**	**Q5(most deprived)**	**City^**b**^**	**Q1 (%)**	**Q5 (%)**	**Q5–Q1 (% units)**
Dresden	1.00 (ref)	1.07 (0.95–1.21)	1.00 (0.89–1.12)	1.10 (0.99–1.23)	**1.18 (1.06–1.32)**	**0.82 (0.75–0.90)**	51.5	47.2	−4.3
Erfurt	1.00 (ref)	0.94 (0.81–1.08)	0.92 (0.81–1.05)	1.02 (0.89–1.17)	0.99 (0.86–1.14)	1.01 (0.89–1.13)	47.9	48.1	0.2
Regensburg	1.00 (ref)	0.99 (0.93–1.06)	1.01 (0.95–1.08)	0.99 (0.92–1.06)	1.04 (0.98–1.11)	NA	51.8	50.7	−1.1

*Ref, Reference; Q, quintile; NA, not applicable as the city Regensburg was included in the quintiles; Significant hazard ratios (p < 0.05) are printed in bold*.

a *adjusted for age, sex, period, cancer site, cancer grade, cancer stage, and surgery*.

b *for the cancer registries Dresden and Erfurt, the cities Dresden and Erfurt were classified separately, as they would otherwise dominate the classification of the quintiles. The deprivation value for Dresden lies between Q1 and Q2 in 2006 and in Q2 in 2010. For Erfurt, it lies in Q1 in 2006 and in Q2 in 2010*.

c *five-year overall survival derived from the direct adjusted survival curves from the adjusted Cox proportional hazard regression model*.

## Discussion

In this first study on the association between socioeconomic deprivation on municipality level and survival of CRC patients in Germany, we showed that patients living in the most deprived areas had a 4.8% units lower 5-year survival compared to patients living in the least deprived areas. Adjustment for differences in patient and tumor characteristics reduced the observed difference to 2.7% units. Results indicate that stratification by treatment factors might weaken the association for patients with stage II and III rectal cancer, but were not consistent. Overall, inequalities were more pronounced in patients with lower cancer stage, with rectal cancer, in the most recent calendar period and were only observed with longer follow-up time. We furthermore observed highest survival in the second least deprived group, which disappeared after excluding patients with residence in the city of Dresden.

Socioeconomic inequalities in CRC survival have been reported in several countries with and without universal health insurance system (4, 5). For example, studies from Canada, the United Kingdom (UK) (20–23), the Netherlands (24), France (25) and Germany (9–11) reported differences in 5-year relative survival between the least and most deprived areas of 5–8% units, which is similar to our effect size of 4.8% units for overall survival. In adjusted analyses, we did not observe a gradient across deprivation groups and the effect size decreased to 2.7% units (corresponding to a 11% higher hazard of death), which is mostly smaller than in adjusted analyses from other countries (10–80% higher hazard of death) (5). Nonetheless, these socioeconomic inequalities warrant further attention, especially as our results indicate an increase of these inequalities over time.

To overcome these socioeconomic inequalities in CRC survival the underlying reasons must be disclosed. Stage was found to be associated with deprivation or explained deprivation-associated differences at least partly in some [e.g., UK (26, 27), Switzerland (28) and New Zealand (29)] but not all previous studies from other countries [e.g., Canada (30), Australia (31), and England (32)]. As in the previous study from Germany (10), survival disparities could not be explained by differences in the stage distribution in our study. Another factor that might explain survival disparities are differences in CRC screening participation rates, as screening-detected CRCs were found to have a better prognosis, even after adjustment for stage (33). Lower participation rates in more deprived areas were reported in various countries (14). In Germany, opportunistic screening by stool test or, since 2002, by screening colonoscopy has been offered. Socioeconomic differences in screening uptake have not been investigated in detail, but regional variations have been found (17, 34). Including data on screening colonoscopy participation rate on district level in our analyses, we could show that adjustment for this factor had only marginal effect on the survival disparities across deprivation groups.

Differences in the provision of cancer care might be another potential cause for socioeconomic inequalities in cancer survival. Previous studies from other countries have reported that patients with lower socioeconomic status or living in more deprived areas had a lower chance to undergo tumor(5) and liver metastasis resection, (35) had a higher risk of getting a permanent stoma, (5) underwent less often laparoscopy (compared to open resection), (36) had less often ≥12 lymph nodes examined, (37) received less often neoadjuvant and adjuvant treatments (5) and were more likely to have a delayed initiation of chemotherapy (38). In our study, adjustment for surgery did not weaken socioeconomic survival disparities. Due to limited availability of data on provided systemic treatments, we could not investigate potential inequalities in the use of neoadjuvant and adjuvant treatments. However, when restricting the patient group to patients who underwent specific treatments, which were recommended according to treatment guidelines, survival differences between the least deprived and the second most deprived and most deprived group remained in patients with stage I and stage II and III CRC and in patients with stage III colon cancer. For stage II and III rectal cancer patients, treatment might affect survival disparities. However, as the sample size was overall too small to come to a conclusion, further studies on socioeconomic differences in CRC care and their impact on survival disparities in Germany are highly needed.

Differences in lifestyle factors and medical history across deprivation groups are another possible cause of socioeconomic inequalities in cancer survival. For example, smoking (39, 40) and comorbidity (32) have been found to be associated with socioeconomic measures and are a prognostic factor for CRC (41, 42). However, as in most registry-based analyses, due to lack of data, we could not investigate these factors. Results from cohort and registry-linkage studies suggest that these factors might partly explain socioeconomic survival disparities (43, 44) and, thus, further investigation of these factors is needed.

In addition to the survival disadvantage in most deprived areas, we observed a survival advantage in the second least deprived areas, which could be attributed to a better survival in the city of Dresden. While a previous study could not find a general difference in cancer survival between urban and rural areas in Germany, (45) patients living in large cities might have a better access to cancer care and, thus, better cancer survival (46). However, we did not find a survival advantage for patients living in the cities Erfurt and Regensburg (data not shown for Regensburg). Furthermore, in region-specific analyses, we observed differences in the association of deprivation with survival across the cancer registries, which need further clarification. Thus, research on general regional variations in cancer survival in Germany should be conducted to better understand regional as well as deprivation-associated inequalities.

Some limitations of our study should be considered in the interpretation of the results. A main limitation of our study is that we could not account for socio-economic differences in general mortality, as we neither had information on cause of death nor life tables by deprivation quintile. While the finding that disparities were largest with longer follow-up and in stage I patients might indicate confounding by differences in general mortality, the stronger association in the younger age group speaks against it (47).

Another limitation of our study is that we cannot distinguish whether individual socioeconomic status or area-based deprivation contributed most to the observed inequalities, as these measure are highly correlated (12). Compared to the previous analysis on the association of area-based deprivation and survival in Germany, (10) we were able to use a small-area measure of deprivation on municipality instead of district level. Nonetheless, with a median population of ≈2,200 residents per municipality, the deprivation measure we used is still a very limited proxy for individual socioeconomic status. Furthermore, interpreting associations from area-based analyses as proxy for patient-level measures can be subject to the so-called ecologic fallacy (48). Further studies using individual level data (ideally together with area-based data) are highly needed to disentangle the association of individual and area-based socioeconomic deprivation and cancer survival. However, health care interventions in Germany would be targeted on area-level, as data on the individual socioeconomic status of a cancer patient would not be available for interventions due to data protection laws. Thus, results from areas-based studies are nonetheless useful.

Due to data availability, we were only able to include data from three population-based clinical cancer registries. While these registries cover more deprived as well as more affluent areas in Germany, an investigation including more cancer registries is highly desirable and will be possible in the future, as nationwide clinical cancer registration is currently being implemented in Germany (49). A further limitation is the lack of data on small-area screening utilization rates and on mode of detection of the cancer. We were only able to adjust for screening utilization rates on district level, which provides a very rough evaluation of the impact of differences in screening utilization on socioeconomic inequalities in cancer survival. As it has been shown that in many countries patients living in more deprived areas are less likely to participate in CRC screening, (14) studies using data on individual or small-area level are highly needed to come to a final conclusion with respect to the impact of screening on socio-economic differences in cancer survival in Germany.

The main strength of our study was the use of a well-established small-area level measure of deprivation (median population: ≈2,200 residents), which has a mostly comparable resolution to area-based indices from other countries [e.g., England (50)]. Furthermore, by using data from clinical cancer registries we had more complete information on stage than in the previous investigation (10) and could for the first time investigate treatment factors. Despite including only three regions in Germany, with a sample size of 38,130 CRC patients, we had sufficient power to detect even weak socioeconomic inequalities.

To conclude, we found socioeconomic inequalities in survival after CRC in Germany with patients living in the most deprived areas having worse survival than patients living in the least deprived areas. These survival disparities were strongest in more recently diagnosed patients, in patients with rectal cancer and stage I cancers and could not be explained by socioeconomic differences in stage distributions and in screening uptake rates. Whether these disparities can be explained by differences in cancer care could not be finally evaluated. As cancer survival should not depend on the socioeconomic status of the patient or the socioeconomic deprivation of the place of residence of the patient, interventions targeted to patients living in the most deprived areas in Germany are needed. However, while this study provides first insights into the underlying reasons for socioeconomic inequalities in cancer survival, further studies are needed that extend the investigation of determinants and disentangle associations with cancer-specific and general mortality.

## Data Availability Statement

The data analyzed in this study was obtained from the clinical cancer registries but restrictions apply to the availability of these data, which were used under license for the current study, and so are not publicly available. Data are however available from the authors upon reasonable request and with permission of the cancer registries.

## Ethics Statement

The studies involving human participants were reviewed and approved by Ethics committee of the medical faculty Heidelberg. Written informed consent to participate in this study was provided by the participants' legal guardian/next of kin.

## Author Contributions

LJ, RP, BH, and HB: designed research (project conception, development of overall research plan, and study oversight). LJ, GB, IF, WM, and MG: conducted research (hands-on conduct of the experiments and data collection). LJ: analyzed data or performed statistical analysis. LJ and HB: wrote paper. LJ and HB: had primary responsibility for final content. All authors read and approved the final manuscript.

## Conflict of Interest

The authors declare that the research was conducted in the absence of any commercial or financial relationships that could be construed as a potential conflict of interest.

## Members of the German Cancer Survival Working Group

Mark Möller, Karin Jäger (Cancer Registry of Bavaria, Regional Center Erlangen-Nürnberg), Carmen Werner (Clinical Cancer Registry Dresden), Paul Strecker (Clinical Cancer Registry Thuringia, Regional Center Erfurt), Michael Gerken (Cancer Registry of Bavaria, Regional Center Regensburg), Karla Geiss, Dorothee Twardella, Martin Meyer (Cancer Registry of Bavaria), Andrea Eberle, Sabine Luttmann (Cancer Registry of Bremen), Heide Wilsdorf-Köhler, Roland Stabenow (Cancer Registry of Berlin and the New Federal States), Cynthia Erb, Alice Nennecke, Stefan Hentschel (Hamburg Cancer Registry), Eunice Sirri, Joachim Kieschke (Cancer Registry of Lower Saxony), Bernd Holleczek (Saarland Cancer Registry), Katharina Emrich (Cancer Registry of Rhineland-Palatinate), Hiltraud Kajüter, Volkmar Mattauch (Cancer Registry of North Rhine-Westphalia), Alexander Katalinic, Ron Pritzkuleit, Johann Mattutat (Cancer Registry of Schleswig-Holstein), Volker Arndt (Cancer Registry of Baden-Württemberg), Werner Maier, Lars Schwettmann (Institute of Health Economics and Health Care Management, Helmholtz Zentrum München), Hermann Brenner, Gundula Behrens, Lina Jansen and Isabelle Finke (German Cancer Research Center).
