# Supplementary material for: Area-Based Socioeconomic Inequalities in Colorectal Cancer Survival in Germany: Investigation Based on Population-Based Clinical Cancer Registration
Source: Front Oncol. 2020 May 29;10:857. doi: 10.3389/fonc.2020.00857 (PMC7326086; doi:10.3389/fonc.2020.00857)
Supplement: Supplementary file 1 [file Data_Sheet_1.docx]

Supplementary Material

***Methods – Multiple imputation***

Multivariate imputation by chained equation was conducted separately for each cancer registry. For each registry, 30 imputed datasets were created using 30 iterations. The imputation model included the factors age, sex, year of diagnosis, cancer site, number of colorectal cancer diagnosis, T stage, N stage, M stage, UICC stage, grade, surgery, chemotherapy, radiotherapy, number of examined lymph nodes, overall and regional deprivation score, vital status and the Nelson-Estimator for the follow-up time.^1^ Model convergence was checked graphically and the distribution of the factors were compared before and after imputation (Supplementary Table 2).

*References*

1. White IR, Royston P. Imputing missing covariate values for the Cox model. *Stat Med* 2009;**28**(15):1982-98.


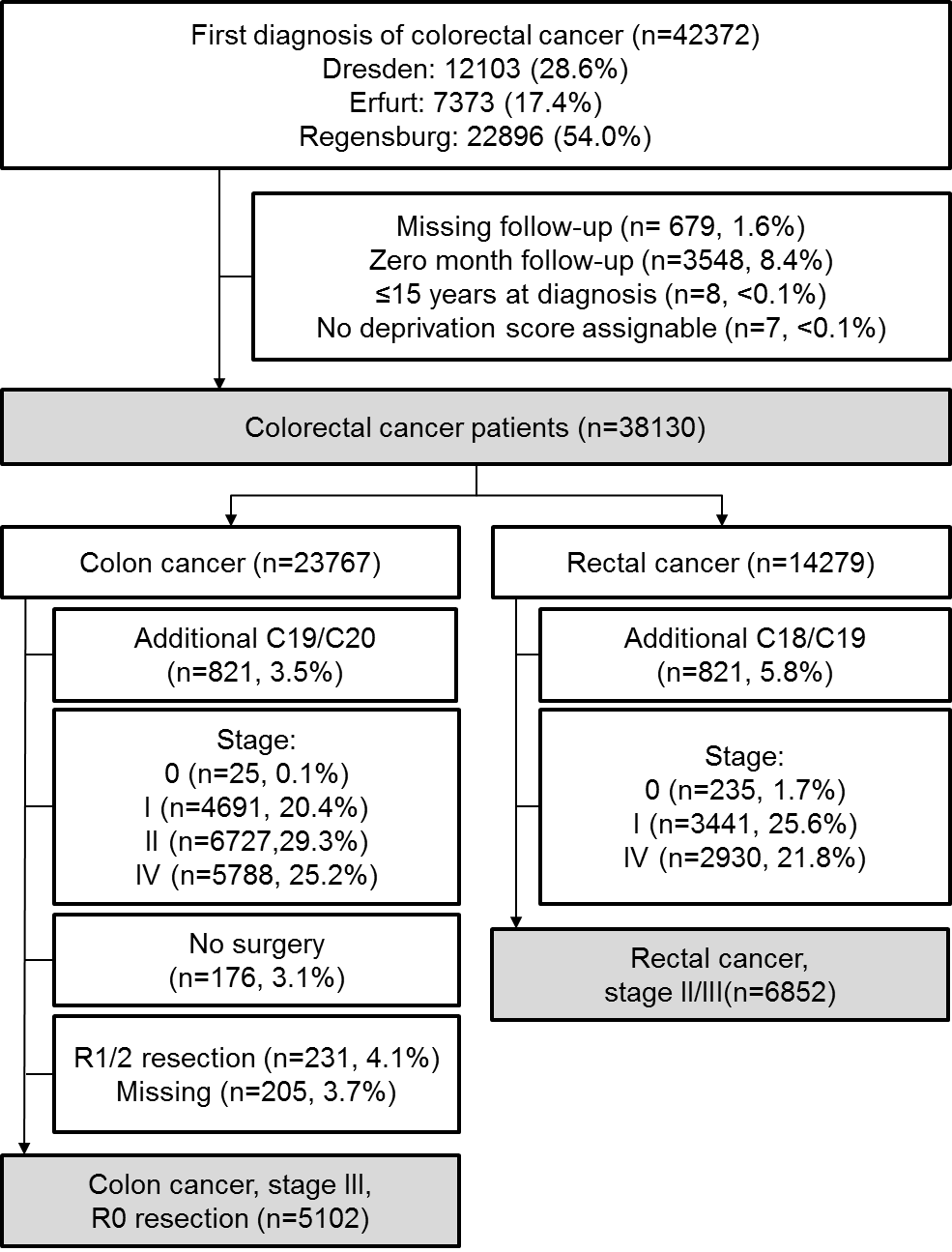


**Supplementary Figure 1.** Flow Chart illustrating the inclusion/exclusions of patients for the main analyses and for subgroup analyses


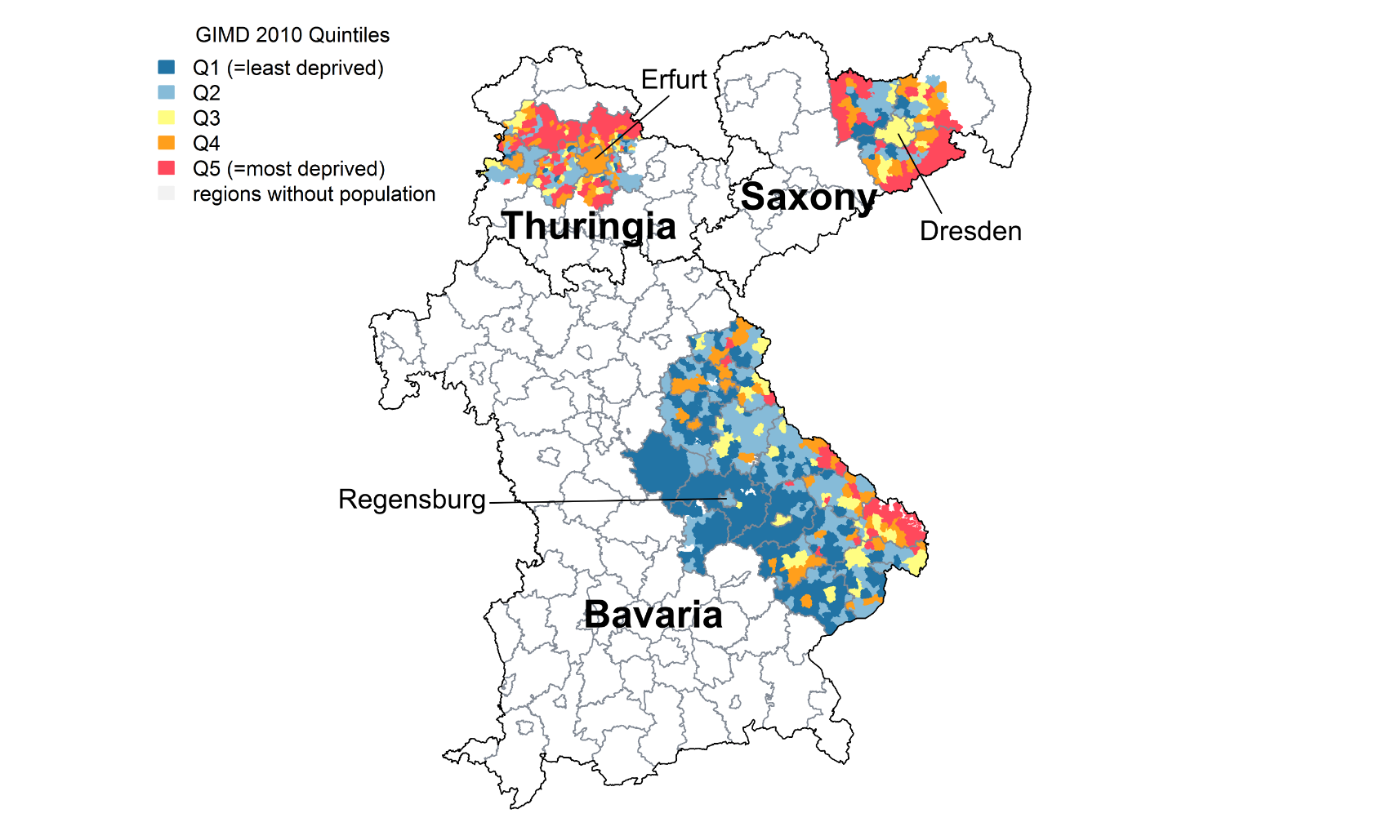


**Supplementary Figure 2.** Classification of included municipalities in Thuringia, Bavaria and Saxony by the socioeconomic deprivation score in 2010.

**Supplementary Table 1** Distribution of factors in the total study population before and after multiple imputation

| **Factor** | **Before Imputation** | **After Imputation** |
| --- | --- | --- |
| **Stage** |  |  |
| I | 21.6% | 22.8% |
| II | 27.1% | 26.4% |
| III | 28.0% | 26.7% |
| IV | 23.0% | 24.1% |
| **Grading** |  |  |
| Low | 73.8% | 73.9% |
| High | 26.2% | 26.1% |
| **Chemotherapy** |  |  |
| No | 56.2% | 56.3% |
| Yes | 43.8% | 43.7% |
| **Radiotherapy** |  |  |
| No | 83.0% | 82.6% |
| Yes | 17.0% | 17.4% |

**Supplementary Table 2** Adjusted five-year survival estimates by socioeconomic deprivation quintile, overall and stratified by patient and tumor characteristics.

| **Subgroup (Model^a^)** |  | **Deprivation quintile  (Adjusted five-year survival estimate)^a^** | | | | | |
| --- | --- | --- | --- | --- | --- | --- | --- |
|  |  | **Q1  (least deprived)** | **Q2** | **Q3** | **Q4** | **Q5  (most deprived)** |  |
| All (Basic model) |  | 51.0 (49.9-52.1) | 55.7 (54.7-56.7) | 52.0 (50.9-53.0) | 49.8 (48.7-50.9) | 48.5 (47.4-49.6) |  |
| All (+ Stage) |  | 51.7 (50.8-52.6) | 54.4 (53.5-55.3) | 51.5 (50.6-52.4) | 50.7 (49.8-51.6) | 49.0 (48.1-49.9) |  |
| All (+ Surgery) |  | 51.7 (50.7-52.6) | 54.3 (53.4-55.1) | 51.5 (50.6-52.4) | 50.8 (49.9-51.7) | 49.0 (48.1-49.9) |  |
|  |  |  |  |  |  |  |  |
| Exclusion of Dresden^b^ |  | 51.5 (50.5-52.4) | 52.1 (50.9-53.3) | 50.6 (49.5-51.6) | 50.6 (49.7-51.6) | 48.8 (47.9-49.7) |  |
|  |  |  |  |  |  |  |  |
| Male |  | 49.7 (48.5-51.0) | 53.0 (51.8-54.2) | 49.8 (48.6-51.0) | 49.3 (48.1-50.6) | 47.3 (46.1-48.5) |  |
| Female |  | 54.4 (53.0-55.8) | 56.0 (54.6-57.3) | 53.8 (52.4-55.2) | 52.9 (51.5-54.3) | 51.3 (49.9-52.6) |  |
|  |  |  |  |  |  |  |  |
| Age 15-64 years |  | 64.6 (63.1-66.1) | 66.5 (65.0-68.0) | 65.4 (63.8-66.9) | 62.9 (61.4-64.4) | 61.6 (60.0-63.1) |  |
| Age 65+ years |  | 45.2 (44.0-46.4) | 48.1 (47.0-49.2) | 44.9 (43.8-46.0) | 44.7 (43.5-45.9) | 43.2 (42.1-44.4) |  |
|  |  |  |  |  |  |  |  |
| Period 2000-2007 |  | 50.7 (49.5-51.9) | 55.5 (54.4-56.6) | 50.4 (49.2-51.6) | 51.4 (50.2-52.6) | 49.4 (48.3-50.5) |  |
| Period 2008-2015 |  | 53.1 (51.6-54.5) | 52.3 (50.8-53.7) | 53.4 (52.0-54.8) | 49.9 (48.4-51.5) | 48.5 (47.0-50.0) |  |
|  |  |  |  |  |  |  |  |
| Colon^c^ |  | 52.8 (51.6-54.0) | 56.0 (54.8-57.1) | 52.6 (51.5-53.7) | 52.9 (51.7-54.1) | 51.2 (50.0-52.4) |  |
| Rectum^c^ |  | 50.1 (48.5-51.7) | 51.9 (50.4-53.5) | 49.6 (47.9-51.2) | 47.7 (46.2-49.3) | 45.6 (44.1-47.2) |  |
|  |  |  |  |  |  |  |  |
| Stage I |  | 72.3 (70.1-74.4) | 76.9 (75.0-78.7) | 72.7 (70.6-74.7) | 69.8 (67.6-71.8) | 68.8 (66.6-70.9) |  |
| Stage II |  | 62.7 (60.6-64.7) | 67.1 (65.2-68.9) | 65.5 (63.5-67.4) | 64.0 (61.9-65.9) | 59.6 (57.6-61.6) |  |
| Stage III |  | 55.7 (53.6-57.7) | 57.2 (55.2-59.1) | 52.5 (50.4-54.6) | 51.4 (49.2-53.6) | 52.4 (50.3-54.4) |  |
| Stage IV |  | 13.8 (12.3-15.3) | 14.9 (13.3-16.4) | 14.0 (12.5-15.5) | 14.9 (13.3-16.6) | 13.2 (11.8-14.7) |  |
|  |  |  |  |  |  |  |  |
| FU length: 5 years^d^ |  | 52.4 (51.4-53.4) | 53.3 (52.3-54.2) | 51.6 (50.7-52.6) | 51.7 (50.7-52.7) | 49.6 (48.7-50.6) |  |
| FU length: 10 years^d^ |  | 51.8 (50.9-52.7) | 53.6 (52.7-54.5) | 51.7 (50.8-52.6) | 51.1 (50.2-52.1) | 49.0 (48.1-50.0) |  |

FU= Follow-up; Q = quintile; ^a^ Estimated from Cox models with adjustment. The basic model includes an adjustment for sex, age, period, cancer site and cancer grade. In the second model, stage and in the third model, surgery was added. In all stratified analyses, model three was used (after omitting the stratification factor). ^b^ The city of Dresden was the largest city in the covered area (523,058 persons), comprising 13.4% of the total underlying study population. ^c^ Patients with a diagnosis of both colon and rectum/rectosigmoid cancer within three months were excluded. Rectum includes rectal (ICD-10 C20) and rectosigmoid (C19) tumors. ^d^ Follow-up length was restricted to a certain time window. Patients dying after this time were censored at the end of the time window.

**Supplementary Table 3** Association of socioeconomic deprivation and cancer survival when classifying the city of Dresden as separate group, overall and stratified by patient and tumor characteristics.

| **Subgroup (Model^a^)** |  | **Events  (N (%))** |  | |  | | **Deprivation quintile  (Hazard ration (95% confidence interval))** | | | | | | | | |  |
| --- | --- | --- | --- | --- | --- | --- | --- | --- | --- | --- | --- | --- | --- | --- | --- | --- |
|  |  |  | |  | | **Q1  (least deprived)** | | **Q2** | **Q3** | **Q4** | **Q5  (most deprived)** | | **Dresden** | |  |  |
| All (Basic model) |  | 19,227 (50.4) | |  | | 1.00 (ref) | | 0.98 (0.93-1.03) | 1.03 (0.98-1.08) | 1.04 (0.99-1.09) | | **1.08 (1.03-1.13)** | | **0.75 (0.71-0.79)** | | |
| All (+Stage) |  | 19,227 (50.4) | |  | | 1.00 (ref) | | 0.97 (0.92-1.02) | 1.04 (0.99-1.09) | 1.04 (0.99-1.09) | | **1.11 (1.06-1.16)** | | **0.84 (0.79-0.88)** | | |
| All (+ Surgery) |  | 19,227 (50.4) | |  | | 1.00 (ref) | | 0.97 (0.92-1.03) | 1.04 (0.99-1.09) | 1.03 (0.98-1.08) | | **1.11 (1.06-1.16)** | | **0.84 (0.80-0.89)** | | |
|  |  |  | |  | |  | |  |  |  | |  | |  | | |
| Male |  | 11,101 (50.0) | |  | | 1.00 (ref) | | 0.99 (0.92-1.06) | 1.02 (0.96-1.09) | 1.01 (0.95-1.08) | | **1.10 (1.03-1.16)** | | **0.80 (0.74-0.85)** | | |
| Female |  | 8,126 (51.1) | |  | | 1.00 (ref) | | 0.96 (0.88-1.04) | 1.06 (0.98-1.15) | 1.06 (0.98-1.15) | | **1.13 (1.05-1.22)** | | **0.91 (0.84-0.99)** | | |
|  |  |  | |  | |  | |  |  |  | |  | |  | | |
| Age 15-64 years |  | 4,456 (36.8) | |  | | 1.00 (ref) | | 0.98 (0.88-1.10) | 0.98 (0.88-1.08) | 1.09 (0.99-1.20) | | **1.16 (1.05-1.27)** | | **0.84 (0.75-0.94)** | | |
| Age 65+ years |  | 14,771 (56.8) | |  | | 1.00 (ref) | | 0.97 (0.91-1.03) | 1.04 (0.98-1.10) | 1.02 (0.96-1.08) | | **1.07 (1.01-1.13)** | | **0.85 (0.80-0.90)** | | |
|  |  |  | |  | |  | |  |  |  | |  | |  | | |
| Period 2000-2007 |  | 11,615 (63.2) | |  | | 1.00 (ref) | | 0.91 (0.84-0.99) | 1.00 (0.94-1.07) | 0.96 (0.90-1.02) | | 1.03 (0.97-1.10) | | **0.74 (0.68-0.80)** | | |
| Period 2008-2015 |  | 7,612 (38.6) | |  | | 1.00 (ref) | | 1.04 (0.97-1.12) | 1.01 (0.92-1.10) | 1.05 (0.97-1.14) | | 1.06 (0.98-1.16) | | **0.82 (0.74-0.91)** | | |
|  |  |  | |  | |  | |  |  |  | |  | |  | | |
| Colon^b^ |  | 11,559 (50.4) | |  | | 1.00 (ref) | | 0.97 (0.90-1.04) | 1.05 (0.98-1.12) | 0.99 (0.93-1.06) | | **1.06 (1.00-1.13)** | | **0.81 (0.76-0.87)** | | |
| Rectum^b^ |  | 7,201 (50.1) | |  | | 1.00 (ref) | | 0.97 (0.89-1.06) | 1.04 (0.96-1.13) | **1.09 (1.01-1.18)** | | **1.18 (1.09-1.27)** | | **0.90 (0.82-0.98)** | | |
|  |  |  | |  | |  | |  |  |  | |  | |  | | |
| Stage I |  | 2,706 (31.1) | |  | | 1.00 (ref) | | 1.00 (0.86-1.17) | 1.06 (0.92-1.21) | 1.13 (0.99-1.28) | | **1.18 (1.04-1.33)** | | **0.65 (0.56-0.74)** | | |
| Stage II |  | 4,166 (41.3) | |  | | 1.00 (ref) | | 0.95 (0.84-1.07) | 0.94 (0.84-1.05) | 0.95 (0.85-1.05) | | **1.12 (1.02-1.24)** | | **0.73 (0.65-0.81)** | | |
| Stage III |  | 4,791 (47.1) | |  | | 1.00 (ref) | | 1.00 (0.90-1.12) | **1.16 (1.05-1.28)** | **1.16 (1.05-1.27)** | | **1.12 (1.02-1.23)** | | **0.90 (0.81-1.00)** | | |
| Stage IV |  | 7,564 (82.3) | |  | | 1.00 (ref) | | 0.96 (0.88-1.04) | 0.99 (0.91-1.07) | 0.96 (0.88-1.04) | | 1.02 (0.95-1.10) | | 0.98 (0.90-1.07) | | |
|  |  |  | |  | |  | |  |  |  | |  | |  | | |
| FU length: 3 months^c^ |  | 2,426 (6.4) | |  | | 1.00 (ref) | | 0.99 (0.86-1.15) | 1.15 (1.00-1.32) | 1.08 (0.94-1.23) | | 1.07 (0.94-1.22) | | 1.05 (0.91-1.22) | | |
| FU length: 1 year^c^ |  | 6,503 (17.1) | |  | | 1.00 (ref) | | 1.04 (0.95-1.13) | 1.05 (0.96-1.14) | 1.00 (0.92-1.08) | | 1.03 (0.95-1.12) | | 0.97 (0.88-1.06) | | |
| FU length: 5 years^c^ |  | 15,649 (41.0) | |  | | 1.00 (ref) | | 1.00 (0.95-1.07) | **1.07 (1.01-1.13)** | 1.03 (0.98-1.08) | | **1.12 (1.06-1.17)** | | **0.91 (0.86-0.97)** | | |
| FU length: 10 years^c^ |  | 18,630 (48.9) | |  | | 1.00 (ref) | | 0.99 (0.94-1.05) | 1.03 (0.98-1.09) | 1.03 (0.98-1.08) | | **1.11 (1.06-1.17)** | | **0.87 (0.82-0.92)** | | |

FU= Follow-up; ref = Reference; Q = quintile; Na = not applicable; Significant hazard ratios (p<0.05) are printed in bold;

^a^ For the overall analyses, three models were used. The basic model includes an adjustment for sex, age, period, cancer site and cancer grade. In the second model, stage and in the third model, surgery was added. In all stratified analyses, model three was used (after omitting the stratification factor).

^b^ Patients with a diagnosis of both colon and rectum cancer within three months were excluded. Rectum includes rectal (ICD-10 C20) and rectosigmoid (C19) tumors.

^c^ Follow-up length was restricted to a certain time window. Patients dying after this time were censored at the end of the time window.

**Supplementary Table 4** Association of socioeconomic deprivation and cancer survival, overall and stratified by patient and tumor characteristics with additional adjustment for screening colonoscopy uptakes rates on district level

| **Subgroup** |  | **Deprivation quintile  (Hazard ration (95% confidence interval))^a^** | | | | | |
| --- | --- | --- | --- | --- | --- | --- | --- |
|  |  | **Q1  (least deprived)** | **Q2** | **Q3** | **Q4** | **Q5  (most deprived)** |  |
| All |  | 1.00 (ref) | **0.88 (0.83-0.92)** | 1.00 (0.96-1.05) | 1.01 (0.96-1.06) | **1.09 (1.04-1.15)** |  |
|  |  |  |  |  |  |  |  |
| Exclusion of Dresden^b^ |  | 1.00 (ref) | 0.97 (0.92-1.03) | 1.03 (0.98-1.09) | 1.03 (0.98-1.08) | **1.11 (1.06-1.16)** |  |
|  |  |  |  |  |  |  |  |
| Male |  | 1.00 (ref) | **0.86 (0.81-0.92)** | 0.98 (0.92-1.05) | 0.99 (0.93-1.05) | **1.08 (1.01-1.15)** |  |
| Female |  | 1.00 (ref) | **0.90 (0.84-0.97)** | 1.04 (0.97-1.13) | 1.04 (0.96-1.13) | **1.13 (1.04-1.21)** |  |
|  |  |  |  |  |  |  |  |
| Age 15-64 years |  | 1.00 (ref) | **0.89 (0.80-0.99)** | 0.98 (0.89-1.09) | 1.10 (0.99-1.21) | **1.18 (1.07-1.30)** |  |
| Age 65+ years |  | 1.00 (ref) | **0.88 (0.83-0.93)** | 1.00 (0.95-1.06) | 0.99 (0.93-1.04) | **1.05 (1.00-1.11)** |  |
|  |  |  |  |  |  |  |  |
| Period 2000-2007 |  | 1.00 (ref) | **0.83 (0.78-0.88)** | 1.01 (0.95-1.08) | 0.97 (0.91-1.03) | 1.05 (0.99-1.11) |  |
| Period 2008-2015 |  | 1.00 (ref) | 0.97 (0.89-1.05) | 0.98 (0.90-1.06) | 1.08 (1.00-1.17) | **1.17 (1.08-1.27)** |  |
|  |  |  |  |  |  |  |  |
| Colon^c^ |  | 1.00 (ref) | **0.85 (0.80-0.91)** | 1.00 (0.94-1.07) | 0.96 (0.90-1.02) | 1.03 (0.97-1.10) |  |
| Rectum^c^ |  | 1.00 (ref) | **0.91 (0.84-0.98)** | 1.02 (0.94-1.11) | 1.08 (0.99-1.17) | **1.19 (1.10-1.29)** |  |
|  |  |  |  |  |  |  |  |
| Stage I |  | 1.00 (ref) | **0.84 (0.73-0.96)** | 0.99 (0.86-1.13) | 1.11 (0.97-1.26) | **1.17 (1.03-1.33)** |  |
| Stage II |  | 1.00 (ref) | **0.78 (0.70-0.87)** | 0.86 (0.78-0.96) | 0.89 (0.80-1.00) | 1.07 (0.97-1.19) |  |
| Stage III |  | 1.00 (ref) | 0.93 (0.84-1.03) | **1.14 (1.03-1.26)** | **1.16 (1.05-1.28)** | **1.14 (1.03-1.25)** |  |
| Stage IV |  | 1.00 (ref) | 0.93 (0.85-1.00) | 0.99 (0.92-1.07) | 0.94 (0.86-1.02) | 1.01 (0.93-1.09) |  |
|  |  |  |  |  |  |  |  |
| FU length: 3 months^d^ |  | 1.00 (ref) | 0.98 (0.85-1.12) | 1.10 (0.96-1.25) | 1.05 (0.92-1.21) | 1.06 (0.92-1.21) |  |
| FU length: 1 year^d^ |  | 1.00 (ref) | 0.96 (0.89-1.05) | 1.02 (0.94-1.11) | 0.96 (0.88-1.04) | 1.00 (0.92-1.09) |  |
| FU length: 5 years^d^ |  | 1.00 (ref) | **0.94 (0.89-0.99)** | 1.03 (0.98-1.09) | 1.00 (0.95-1.06) | **1.10 (1.04-1.16)** |  |
| FU length: 10 years^d^ |  | 1.00 (ref) | **0.90 (0.86-0.95)** | 1.00 (0.95-1.05) | 1.00 (0.95-1.05) | **1.10 (1.05-1.15)** |  |

Q = quintile; ref = Reference; Significant hazard ratios (p<0.05) are printed in bold;

^a^ Adjustment for sex, age, period, cancer site, cancer stage, grade, surgery and utilization proportion of screening colonoscopies in the district of the place of residence of the patient at diagnosis. In stratified analyses, the stratification factors was omitted from the model.

^b^ The city of Dresden was the largest city in the covered area (523,058 persons), comprising 13.4% of the total underlying study population.

^c^ Patients with a diagnosis of both colon and rectum cancer within three months were excluded. Rectum includes rectal (ICD-10 C20) and rectosigmoid (C19) tumors. ^d^ Follow-up length was restricted to a certain time window. Patients dying after this time were censored at the end of the time window.

**Supplementary Table 5** Adjusted five-year survival estimates by socioeconomic deprivation quintile for subgroups according to received treatment

| **Subgroup** |  | **Deprivation quintile  (Five-year survival estimate)^a^** | | | | | |
| --- | --- | --- | --- | --- | --- | --- | --- |
|  |  | **Q1  (least deprived)** | **Q2** | **Q3** | **Q4** | **Q5  (most deprived)** |  |
| Stage I |  | 74.3 (72.2-76.3) | 78.7 (76.9-80.4) | 74.6 (72.5-76.5) | 71.5 (69.4-73.6) | 70.7 (68.5-72.7) |  |
| + with surgery |  | 75.3 (73.2-77.3) | 79.9 (78.1-81.5) | 75.5 (73.4-77.3) | 72.4 (70.2-74.4) | 71.5 (69.3-73.6) |  |
|  |  |  |  |  |  |  |  |
| Stage II-III |  | 60.6 (59.1-62.0) | 63.4 (62.1-64.8) | 59.9 (58.5-61.3) | 58.2 (56.7-59.7) | 56.7 (55.2-58.1) |  |
| + surgery |  | 61.9 (60.4-63.3) | 64.6 (63.2-66.0) | 61.3 (59.8-62.7) | 59.5 (58.0-61.0) | 58.1 (56.6-59.5) |  |
| + chemotherapy |  | 65.8 (63.4-68.0) | 68.1 (65.8-70.2) | 64.0 (61.7-66.2) | 62.4 (60.0-64.6) | 61.5 (59.1-63.7) |  |
|  |  |  |  |  |  |  |  |
| Stage III colon cancer |  | 59.2 (56.4-61.9) | 60.9 (58.3-63.4) | 55.4 (52.7-58.1) | 54.5 (51.6-57.4) | 55.7 (52.9-58.4) |  |
| + with R0 surgery |  | 61.6 (58.7-64.4) | 63.1 (60.4-65.7) | 58.1 (55.2-60.9) | 57.9 (54.8-60.9) | 59.0 (56.0-61.9) |  |
| + with chemotherapy |  | 68.6 (64.7-72.1) | 71.7 (68.1-74.9) | 64.1 (60.1-67.8) | 65.8 (61.7-69.6) | 64.2 (60.2-67.9) |  |
|  |  |  |  |  |  |  |  |
| Stage II&III rectal cancer |  | 55.7 (53.0-58.2) | 57.8 (55.2-60.2) | 54.4 (51.8-57.0) | 52.2 (49.6-54.7) | 50.8 (48.3-53.3) |  |
| + radiotherapy |  | 60.1 (56.5-63.4) | 64.3 (60.7-67.6) | 58.1 (54.3-61.7) | 55.6 (51.8-59.1) | 56.4 (52.6-60.0) |  |
| + neoadj. radiotherapy |  | 60.9 (56.0-65.3) | 66.0 (60.9-70.6) | 60.3 (54.8-65.2) | 61.3 (55.8-66.3) | 56.1 (50.4-61.3) |  |

^a^ Adjusted for age, sex, period, cancer site (for overall analyses), cancer grade and cancer stage (for stage II&III analyses)

**Supplementary Table 6** Association of socioeconomic deprivation and cancer survival for subgroups according to received treatment when classifying the city of Dresden as separate group

| **Subgroup** |  | **Events  (N (%))** |  | **Deprivation quintile  (Hazard ration (95% confidence interval))^a^** | | | | | |  |
| --- | --- | --- | --- | --- | --- | --- | --- | --- | --- | --- |
|  |  |  |  | **Q1  (least deprived)** | **Q2** | **Q3** | **Q4** | **Q5  (most deprived)** | **Dresden** | |
| Stage I |  | 2,706 (31.1) |  | 1.00 (ref) | 1.00 (0.86-1.17) | 1.06 (0.93-1.22) | **1.14 (1.01-1.29)** | **1.19 (1.05-1.34)** | **0.65 (0.57-0.75)** | |
| + with surgery |  | 2,524 (30.0) |  | 1.00 (ref) | 1.00 (0.85-1.16) | 1.07 (0.93-1.23) | **1.15 (1.01-1.31)** | **1.20 (1.06-1.36)** | **0.64 (0.55-0.74)** | |
|  |  |  |  |  |  |  |  |  |  | |
| Stage II&III |  | 8,957 (47.1) |  | 1.00 (ref) | 0.98 (0.91-1.06) | 1.08 (1.00-1.16) | **1.09 (1.02-1.17)** | **1.15 (1.07-1.23)** | **0.82 (0.76-0.88)** | |
| + surgery |  | 8,321 (42.9) |  | 1.00 (ref) | 0.99 (0.91-1.08) | 1.07 (1.00-1.16) | **1.09 (1.01-1.17)** | **1.15 (1.07-1.23)** | **0.81 (0.75-0.88)** | |
| + chemotherapy |  | 3,640 (38.4) |  | 1.00 (ref) | 0.97 (0.85-1.10) | 1.07 (0.96-1.20) | **1.13 (1.02-1.26)** | **1.17 (1.05-1.30)** | 0.90 (0.79-1.02) | |
|  |  |  |  |  |  |  |  |  |  | |
| Stage III colon cancer |  | 2,726 (47.4) |  | 1.00 (ref) | 1.03 (0.89-1.20) | **1.21 (1.06-1.38)** | **1.17 (1.03-1.33)** | 1.13 (0.99-1.28) | **0.87 (0.75-0.99)** | |
| + with R0 surgery |  | 2,280 (47.7) |  | 1.00 (ref) | 1.05 (0.90-1.23) | **1.20 (1.04-1.38)** | 1.14 (0.99-1.31) | 1.10 (0.96-1.26) | 0.86 (0.74-1.00) | |
| + with chemotherapy |  | 1,142 (36.3) |  | 1.00 (ref) | 0.97 (0.78-1.21) | 1.19 (0.98-1.44) | 1.11 (0.92-1.35) | 1.18 (0.98-1.43) | 0.87 (0.71-1.08) | |
|  |  |  |  |  |  |  |  |  |  | |
| Stage II&III rectal cancer |  | 3,102 (45.3) |  | 1.00 (ref) | 0.99 (0.86-1.13) | 1.08 (0.96-1.23) | **1.12 (1.00-1.26)** | **1.18 (1.05-1.32)** | **0.86 (0.75-0.98)** | |
| + radiotherapy |  | 1,738 (40.1) |  | 1.00 (ref) | 0.96 (0.81-1.14) | 1.06 (0.91-1.25) | **1.16 (1.00-1.34)** | 1.15 (0.99-1.34) | **0.80 (0.66-0.98)** | |
| + neoadj. radiotherapy |  | 751 (30.2) |  | 1.00 (ref) | 0.88 (0.69-1.12) | 0.98 (0.77-1.23) | 0.95 (0.76-1.19) | 1.13 (0.91-1.41) | 0.76 (0.56-1.04) | |

Ref = reference; Q = quintile; Significant hazard ratios (p<0.05) are printed in bold;

^a^ Adjusted for age, sex, period, cancer site (for combined analysis of colon and rectal cancer), cancer grade and cancer stage (for stage II&III analyses).

**Supplementary Table 7** Adjusted five-year survival estimates for each single registry using socioeconomic deprivation quintiles derived within the catchment area

| **Subgroup** |  | **Deprivation quintile  (Five-year survival estimate)^a^** | | | | | |  |
| --- | --- | --- | --- | --- | --- | --- | --- | --- |
|  |  | **Q1  (least deprived)** | **Q2** | **Q3** | **Q4** | **Q5  (most deprived)** | **City^b^** | |
| Dresden |  | 51.5 (49.3-53.6) | 49.7 (47.3-52.0) | 51.5 (49.3-53.7) | 49.0 (47.0-51.1) | 47.2 (45.1-49.3) | 56.3 (55.1-57.5) | |
| Erfurt |  | 47.9 (45.1-50.6) | 49.6 (46.8-52.3) | 50.0 (47.4-52.6) | 47.4 (44.8-49.9) | 48.1 (45.3-50.8) | 47.7 (45.9-49.6) | |
| Regensburg |  | 51.8 (50.5-53.1) | 52.0 (50.7-53.2) | 51.5 (50.2-52.8) | 52.1 (50.8-53.4) | 50.7 (49.5-52.0) | Na | |

Na= not applicable;

^a^ Estimated from Cox models with adjustment for sex, age, period, cancer site, cancer stage, cancer grade and surgery.

^b^ For the cancer registries Dresden and Erfurt, the cities Dresden and Erfurt were classified separately, as they would otherwise dominate the classification of the quintiles. The deprivation value for Dresden lies between Q1 and Q2 in 2006 and in Q2 in 2010. For Erfurt, it lies in Q1 in 2006 and in Q2 in 2010.
